# Suppression and Activation of the Malignant Phenotype by Extracellular Matrix in Xenograft Models of Bladder Cancer: A Model for Tumor Cell “Dormancy”

**DOI:** 10.1371/journal.pone.0064181

**Published:** 2013-05-24

**Authors:** Robert E. Hurst, Paul J. Hauser, Kimberly D. Kyker, Jonathan E. Heinlen, Jason P. Hodde, Michael C. Hiles, Stanley D. Kosanke, Mikhail Dozmorov, Michael A. Ihnat

**Affiliations:** 1 Department of Urology, College of Medicine, Oklahoma University Health Sciences Center, Oklahoma City, Oklahoma, United States of America; 2 Department of Biochemistry and Molecular Biology, College of Medicine, Oklahoma University Health Sciences Center, Oklahoma City, Oklahoma, United States of America; 3 Department of Physiology, College of Medicine, Oklahoma University Health Sciences Center, Oklahoma City, Oklahoma, United States of America; 4 Stephenson Cancer Center, College of Medicine, Oklahoma University Health Sciences Center, Oklahoma City, Oklahoma, United States of America; 5 Department of Pathology, College of Medicine, Oklahoma University Health Sciences Center, Oklahoma City, Oklahoma, United States of America; 6 Department of Pharmaceutical Sciences, College of Pharmacy, Oklahoma University Health Sciences Center, Oklahoma City, Oklahoma, United States of America; 7 Cook Biotech, Inc., West Lafayette, Indiana, United States of America; 8 DormaTarg, Inc., Oklahoma City, Oklahoma, United States of America; 9 Arthritis and Clinical Immunology Research Program, Oklahoma Medical Research Foundation, Oklahoma City, Oklahoma, United States of America; Northwestern University, United States of America

## Abstract

A major problem in cancer research is the lack of a tractable model for delayed metastasis. Herein we show that cancer cells suppressed by SISgel, a gel-forming normal ECM material derived from Small Intestine Submucosa (SIS), in flank xenografts show properties of suppression and re-activation that are very similar to normal delayed metastasis and suggest these suppressed cells can serve as a novel model for developing therapeutics to target micrometastases or suppressed cancer cells. Co-injection with SISgel suppressed the malignant phenotype of highly invasive J82 bladder cancer cells and highly metastatic JB-V bladder cancer cells in nude mouse flank xenografts. Cells could remain viable up to 120 days without forming tumors and appeared much more highly differentiated and less atypical than tumors from cells co-injected with Matrigel. In 40% of SISgel xenografts, growth resumed in the malignant phenotype after a period of suppression or dormancy for at least 30 days and was more likely with implantation of 3 million or more cells. Ordinary Type I collagen did not suppress malignant growth, and tumors developed about as well with collagen as with Matrigel. A clear signal in gene expression over different cell lines was not seen by transcriptome microarray analysis, but in contrast, Reverse Phase Protein Analysis of 250 proteins across 4 cell lines identified Integrin Linked Kinase (ILK) signaling that was functionally confirmed by an ILK inhibitor. We suggest that cancer cells suppressed on SISgel could serve as a model for dormancy and re-awakening to allow for the identification of therapeutic targets for treating micrometastases.

## Introduction

Expression of the malignant phenotype is neither an immediate nor even inevitable consequence of the mutation of tumor suppressor genes or oncogenes. It has long been known that without remodeling the extracellular matrix (ECM), cancer cells are unable to form tumors [1], [2] and that the ECM itself contains elements both antagonistic and agonistic of the malignant phenotype [1]. The importance of the ECM has recently become more apparent as the phenomenon of dormancy of metastatic cells has been recognized [3]–[5], and that the first committed step in metastasis is escape of micrometastatic cells from local inhibitory factors that tend to favor continued dormancy [6], [7]. Also, the discovery that cells with abnormal genomes express tumor-associated antigens in histopathologically normal urothelium [8] or cells with p53 mutations characteristic of the primary tumor are found in histopathologically normal oral mucosa [9] demonstrates that cancer cells can masquerade as normal cells. The suppression of the malignant properties of cells with the potential for tumor formation may well underlie the latency period of primary tumor growth, as well as of delayed local and distal recurrence. Perhaps “dormancy” could contribute to the reason that even with newer “targeted” therapies and billions of dollars in cancer research, overall cancer-specific survival has changed little [10], [11]. Dormant cells have not even been recognized as a potential target until recently [12], and clearly such cells are resistant to conventional chemotherapy because of the very limited efficacy of adjuvant chemotherapy [3], [13]. What is needed is a model system with which to investigate the mechanisms of suppression and activation of cancer cells by the ECM that can also be used to identify and test drugs that target ECM-suppressed cancer cells.

Earlier we demonstrated that the phenotype of bladder cancer cells was radically different in 3-dimensional organotypic culture when grown on a normal extracellular matrix preparation (SISgel) as compared to that observed on a cancer-modulated permissive extracellular matrix preparation (Matrigel) [14]. SISgel is a gel-forming material derived from acellular porcine small intestine submucosa, whereas Matrigel is a basement membrane preparation obtained from a mouse sarcoma [15]. When grown on Matrigel the bladder cancer cells recapitulated the phenotype reported for the original tumor. In sharp contrast, most of the malignant properties were lost when the cells were grown on SISgel [14]. Cell lines derived from papillomas cultured on SISgel formed a layered structure reminiscent of normal urothelium, whereas cell lines derived from higher grade tumors formed a noninvasive layer of cells [14]. These findings suggested that growth of cancer cells on normal ECM could provide a model to investigate the phenomenon of suppression of malignancy by normal ECM and its role in metastasis and recurrence.

In this communication we explored whether the phenotypic suppression seen in organotypic culture of bladder cancer cells on SISgel is also observed *in vivo*. Positive findings support the use of SISgel as a model for investigations of the dormant or suppressed tumor cell phenotype and of mechanisms by which the normal ECM exerts an inhibitory influence on tumorigenesis and metastasis. The findings strongly suggest that interactions of cancer cells with normal ECM play an important role in recurrence and metastasis and further suggest that targeting suppressed cells could represent a heretofore unexploited point of vulnerability in cancer therapy.

## Results

**Figure 1:**
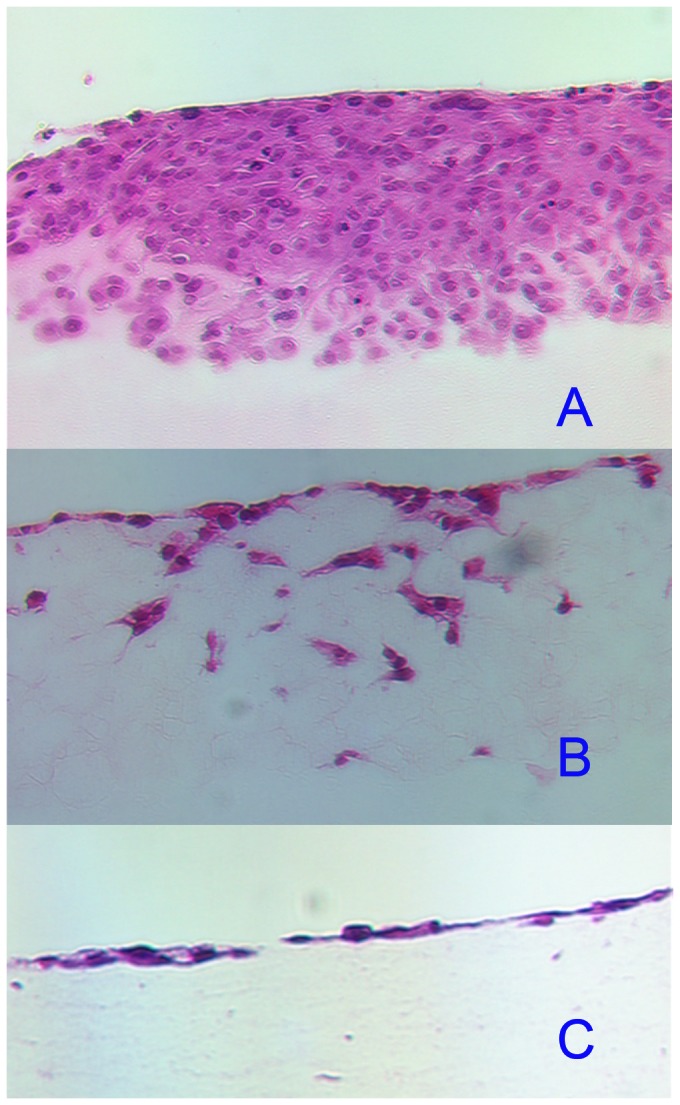
Comparison of the phenotypes of J82 bladder cancer cell lines grown on (A) Matrigel, (B) collagen or (C) SISgel in 3-D culture. Note the loss of invasion and of the more orderly appearance of the cells grown on SISgel. All magnifications are 200X. Images are representative of a minimum of 4 experiments.

The phenotype of one of the aggressive bladder cancer cell lines grown on SISgel, collagen and Matrigel in culture is illustrated in Fig. 1. An invasive, aggressive phenotype on Matrigel and collagen is observed. On Matrigel, the cells invaded, but as a front, as is observed in clinical tumors. On collagen, individual cells invaded, whereas some cells remained at the surface of the gel, and those that invaded had a mesenchymal appearance. The cells grown on Matrigel also showed a range of sizes and nuclear orientations indicative of an aggressive phenotype. This phenotype contrasts markedly with that observed on SISgel, where the cells failed to invade and formed a layer on top of the gel. The cells showed an epithelial phenotype, and the nuclei were more uniform and organized indicative of a normalized phenotype.

**Figure 2:**
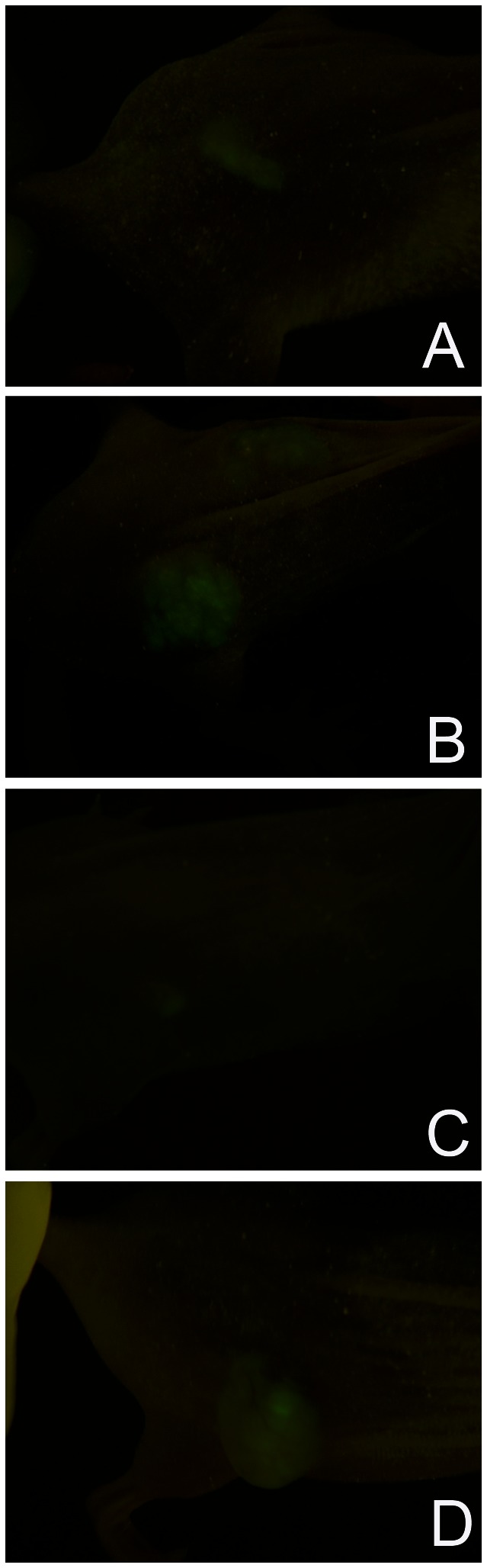
Examples of fluorescent image of GFP-expressing J82 bladder cancer cells in flank xenografts. (A) Cells observed 24 hr after implantation. (B) cells co-injected with Matrigel observed after 60 days showing actively growing tumor. (C) cells co-injected with SISgel observed after 50 days showing cells remain viable but are not growing into a tumor (“glowing spot”). (D) cells co-injected with SISgel that escaped following initial suppression shown 60 days after injection. The cells remained as a glowing spot (see C) for 45 days, then resumed rapid growth as a tumor. Growth was defined as two consecutive increases in size.

We next determined whether the suppression of malignant properties seen in 3-dimensional culture was also observed *in vivo* in flank xenografts. Typical results using the J82 cells are illustrated in Fig. 2. A fluorescence image of cells just after injection is shown in Fig. 2A. The appearance of a tumor that grew from cells co-injected with Matrigel is illustrated in Fig. 2B. Fig. 2C shows the typical result with cells co-injected with SISgel. The cells remain as a flat spot that glows faintly under exciting illumination but which generally does not form a growing tumor and remains visible for weeks. In some fraction of these SISgel xenografts, however, the cells escape from suppression and begin growing as an active tumor, as is illustrated in Fig. 2D. As is shown below, the fraction that escapes to resume active growth depends upon the number of cells injected.

**Figure 3:**
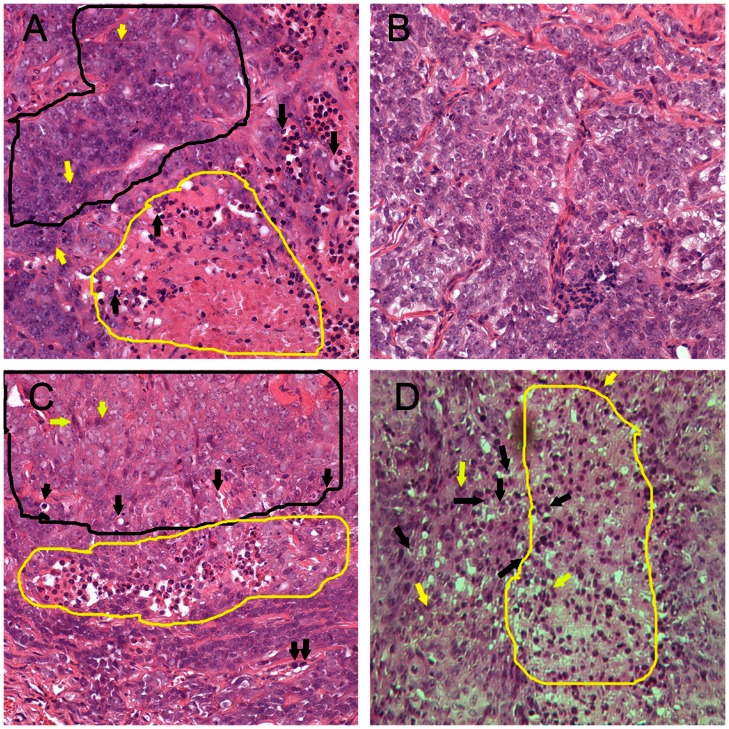
Histopathology of cells from flank xenografts. Features identified histopathologically are shown as follows. Areas of malignant cells are outlined in black, and areas of coagulative necrosis are outlined in yellow. Black arrows illustrate apoptotic bodies. Yellow arrows identify mitotic figures. At the zoom level shown here these are difficult to distinguish but were identified under high power. (A) Cells co-injected with Matrigel that immediately presented a malignant growth pattern. The pathologic description noted large areas of coagulative necrosis with acute inflammation (the small, dense cells are neutrophils) with an area of highly atypical cells revealing marked nuclear pleomorphism, all indicative of high grade neoplasia. (B) Cells co-injected with SISgel that remained in the suppressed or dormant phenotype. This slide was read as cells with moderate cellular atypia and pleomorphism with minimal evidence of coagulative necrosis, mitosis and apoptosis. (C) Cells co-injected with SISgel that initially presented a suppressed phenotype for 8 weeks but then resumed growth in the malignant phenotype. This was read as containing an area of coagulative necrosis with acute inflammation and foci of markedly atypical cells along with prominent mitosis and apoptosis. Some fields contained multinucleated tumor giant cells usually indicative of high-grade neoplasia (D) Cells co-injected with Collagen I demonstrating a malignant growth pattern similar to those illustrated in panels A and C. All images are at 400X.

**Figure 4:**
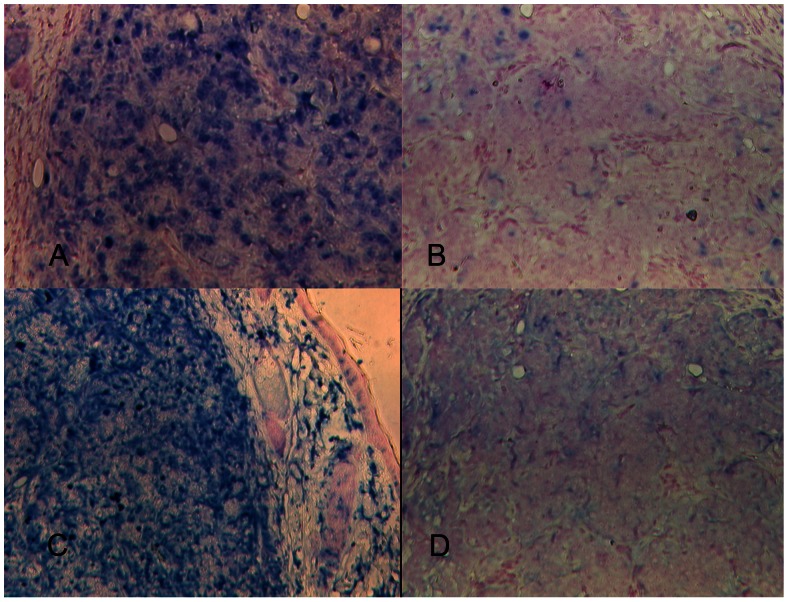
Comparison of Ki67 (proliferation) and vimentin in suppressed cell tissue and actively growing tumors. JB-V cells co-injected with Matrigel and labeled for Ki67 (A) and vimentin (C). JB-V cells co-injected with SISgel and in the suppressed phenotype labeled for Ki67 (B) and vimentin (D). Controls omitting the primary antibody (not shown) were entirely negative for both markers. Images at 200X.

The tumors that formed from cells co-injected with Matrigel, collagen I or that escaped from cells co-injected with SISgel were examined histopathologically together with one of the spots of suppressed fluorescent cells that failed to develop into a growing tumor. These results, presented in Fig.3, confirm that cells are suppressed by SISgel. Fig. 3A shows a typical section from JB-V cells that grew into a tumor following co-injection with Matrigel. The appearance is that of an aggressive tumor with numerous apoptotic bodies and mitotic figures. The cells are markedly pleiomorphic and highly anaplastic, areas of coagulative necrosis are present and most vessels appear abnormal. The histopathology of one of the suppressed patches of cells co-injected with SISgel shown grossly in Fig. 2C is presented in Fig. 3B. In contrast to the actively growing tumors, the cells are more differentiated, less anaplastic or pleiomorphic with normal appearing vessels, very few mitotic figures, and mimal evidence of apoptotic or necrotic areas. Fig. 3C illustrates a section from one of the tumors that grew from SISgel following a period of dormancy of 8 weeks. The tumor is histopathologically similar to Fig. 3A and confirms that when tumors escape from suppression, they resume in an aggressive phenotype. Fig. 3D shows that cells co-injected with collagen also form an aggressive-appearing tumor with characteristics similar to Figs 3A and 3B. Trichrome staining for collagen in all the tissues revealed that by the time these tumors or patches of suppressed cells were harvested, the original collagen in the co-injection medium had vanished (not shown). The only discernible collagen in Fig. 3B was the vessels. Fig. 4 contrasts expression of the proliferation marker, Ki67 and the mesenchymal marker, vimentin in flank xenografts and confirms the histopathologic assessment presented in Fig. 3. As is shown, very few cells in the suppressed cell tissue are in the cell cycle, and there is very little vimentin expression. In contrast, the actively growing tumors were characterized by a high level of expression of both, indicating an aggressively growing phenotype. Tumors from J82 cells were very similar to those presented for JB-V cells in all respects.

**Figure 5:**
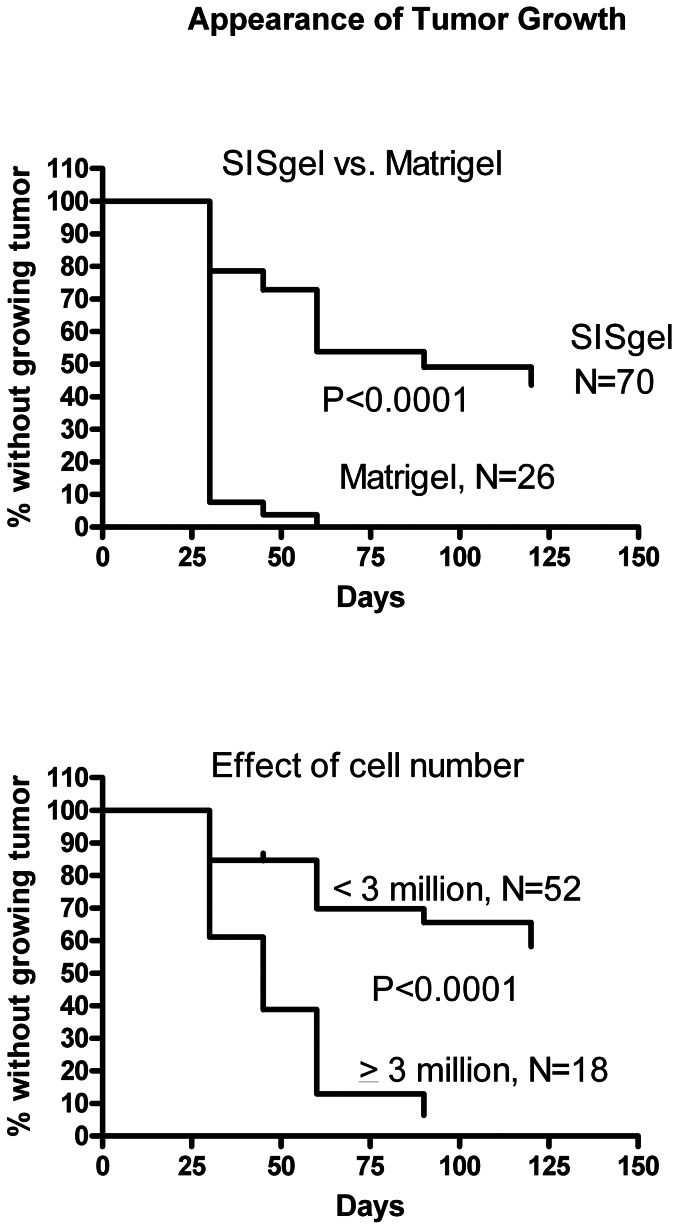
Kaplan-Meier analysis with the endpoint of beginning active growth of a tumor, i.e. survival is defined as the absence of active growth. (A) SISgel vs Matrigel. (B) Three or more million cells implanted vs. fewer than three million cells.

Kaplan-Meier plots of the data are shown in Fig. 5A with an endpoint of beginning tumor growth; that is in this case “survival” is counted as survival of a suppressed phenotype. Because all experiments contained Matrigel positive controls with the same cell numbers in the same proportions as were used for SISgel, Fig. 5A combines the experiments with different cell numbers. When co-injected with Matrigel, cells developed into tumors within 30 days in 100% of animals, even with as few as 250,000 cells. With SISgel, this clearly was not the case, and only about 40% of animals eventually developed growing tumors after a delay of between four and as long as 18 weeks. The difference in behavior between cells co-injected with Matrigel and SISgel was highly statistically significant (p<0.0001). A total of 70 xenografts were prepared from SISgel. Of the 60% of animals in which no tumor developed, many of the implanted cells remained as a green fluorescent spot, although in some cases the spot was no longer discernible by several weeks. In two xenografts in which the implanted cells had apparently disappeared, tumors reappeared in one animal at 12 weeks following the initial injection, while in another animal a growing tumor was clearly forming by 15–16 weeks. Even though at least 95% of cells were fluorescent when prepared, in two cases, re-emergent tumors failed to express GFP as shown by gross examination, and in another, gross examination showed a mosaic of fluorescent and non-fluorescent cells. The re-emergence of the malignant phenotype is dependent on cell number, with more tumors more likely to occur when more than 3 million cells are injected than when fewer than 3 million cells were injected (p<0.0001) (Fig. 5B). There were no discernible differences in the behavior of JB-V and J82 cells (p-values of survival curves were all greater than 0.2) even though the JB-V cells had been selected to be highly metastatic and to grow preferentially in the bladder [16].

We also tested whether the gel co-injected with the tumor cells was dominant over the cancer cell phenotype attained in culture. Table 1 summarizes the growth of tumors according to the ECM on which they were grown in culture as well as which ECM was co-injected into the flank with the tumor cells after growth on ECM in culture. The ECM co-injected into animals with the bladder tumor cells governed whether tumors formed, and the phenotype established by ECM on which the cells were grown was overcome by the co-injected ECM. As expected, cells that were co-injected with Matrigel uniformly formed growing tumors, regardless of how they had been grown in culture. Co-injection with SISgel suppressed bladder cancer cells that had been actively growing and fully expressing the malignant phenotype on Matrigel *in vitro.* Interestingly, cells grown on Matrigel and co-injected with culture medium alone were not capable of tumor formation, further supporting that the local matrix is more important than the phenotype of the cells at the time they were injected. No difference in size, growth rate or gross appearance was detectable between tumors formed from cells that had been grown in the suppressive phenotype on SISgel but were co-injected with Matrigel as compared to cells that had been grown on Matrigel or plastic and were co-injected with Matrigel.

**Table 1:** Tumor growth at 28 days from 3 million cells as a function of ECM preparation on which J82 bladder cancer cells were grown and ECM preparation co-injected with J82 bladder cancer cells.

	Substance co-injected with J82 bladder cancer cells.		
ECM on which cells were grown	SISgel	Matrigel	Culture Medium
Matrigel	0/4	4/4	0/4
SISgel	0/4	4/4	0/4

Because the main component of SISgel is collagen I [17], we also co-injected J82 cells that had been grown on plastic with collagen I. In contrast to SISgel, collagen I tumors grew rapidly; and in eight of 12 animals in three different experiments tumors grew within the timeframe established for Matrigel. In these same experiments none of 12 animals co-injected with SISgel developed tumors. The difference between cells co-injected with collagen and SISgel was highly significant (p = 0.0013), but the difference between cells co-injected with collagen and Matrigel was not (p>0.05).

We also investigated the mechanism for suppression by comparing gene expression and protein expression profiles of cells grown on SISgel with the same cells grown on Matrigel. The microarray studies were essentially uninformative, suggesting that the phenomenon of suppression is not regulated at the gene expression level. Although 243 genes were differentially expressed between J82 cells grown on SISgel vs. Matrigel and 214 were differentially expressed by JBV cells grown under the same conditions, there was very little overlap. Only 11 genes were common to the two lists, and none were consistently differentially expressed across matrices in the same direction by both cell lines. Examination of the ontologies of the gene sets showed that the set of genes differentially expressed by the two cell lines grown on Matrigel or SISgel were different ontologies. We conclude that the lack of a consistent gene expression signature between different cell lines grown on different matrix preparation indicates that the obvious phenotypic differences must result from differential expression at the protein level rather than at the gene expression level of regulation.

**Figure 6:**
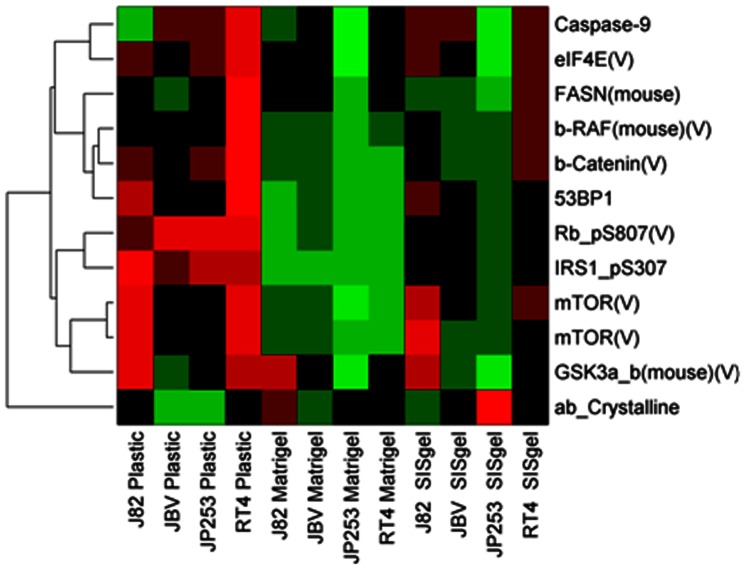
Signature of proteins that are statistically significantly differentially expressed comparing cells from 4 lines grown on plastic, SISgel and Matrigel. The symbol (V) indicates the antibody was validated to show a single band by Western blot. A “p” indicates that the antibody is against a phosphorylated form of the protein, with the amino acid position and identity provided. The two mTOR entries represent duplicates. A complete list of the antibodies used at the time of the analysis is provided as Table S1.

**Figure 7:**
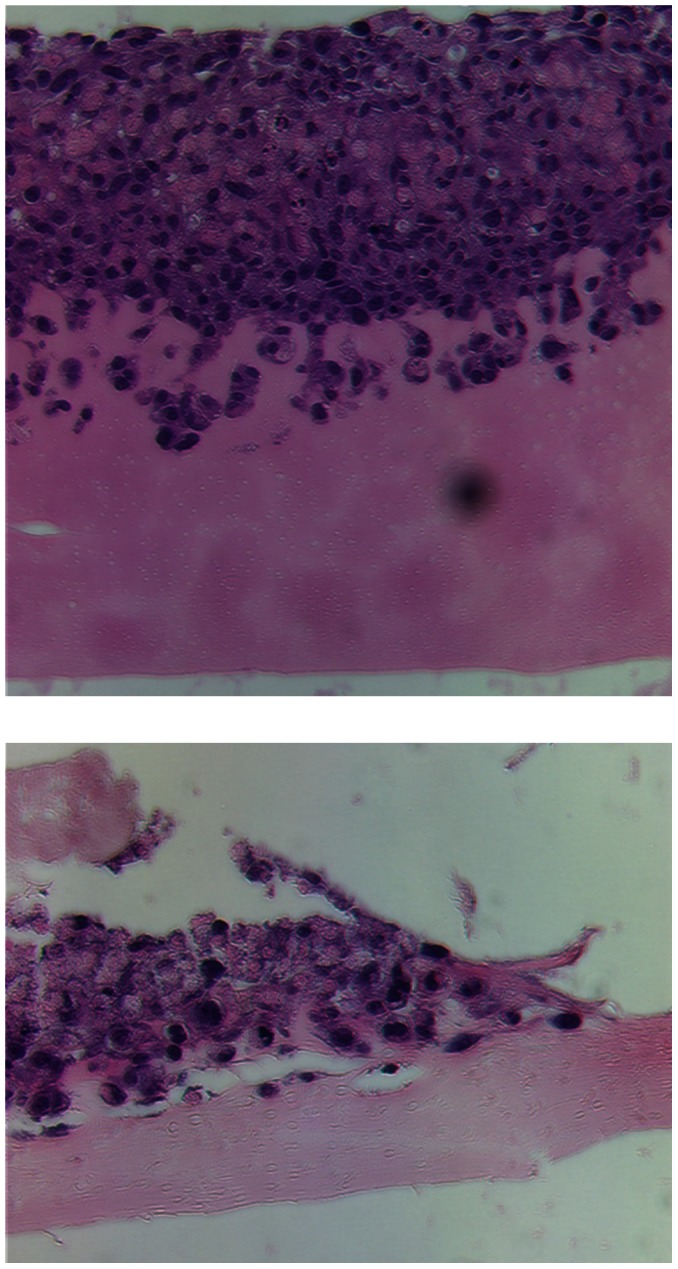
Functional role of ILK signaling in SISgel suppressed cells in culture. J82 cells grown on Matrigel for five days with no ILK inhibitor (top) or with 10 µM QLT0267 ILK inhibitor (bottom panel). Note suppression of invasion. All magnifications are 200X. Images are representative of 3 experiments.

Reverse Phase Protein Array (RPPA) protein analysis for 250 key signaling proteins did identify (Fig. 6A) a consistent signature across all 4 cell lines. Pathway analysis by IPA using the gene names showed the following were highly significantly involved. PI3K/AKT Signaling (p = 1.00×10^−8^, MTOR, GSK3A, GSK3B, TNNB1, EIF4E), Insulin Receptor Signaling (p = 1.70×10^−8^ BRAF, RB1, MTOR, GSK3B, CTNNB1) and ILK Signaling (p = 9.12×10^−8^ MTOR, IRS1, GSK3A, GSK3B, CTNNB1). The functional role of ILK was confirmed using a small molecule inhibitor of ILK, which showed that inhibition of ILK in J82 cells grown on Matrigel produced a noninvasive phenotype very similar to that seen when the cells were grown on SISgel as shown in Fig. 7. The typical invasive tumor-like phenotype is seen in Fig. 7A with J82 cells grown on Matrigel. In contrast, the invasive phenotype is abolished when 10 µM QLT0267 is added to the culture medium of the cells grown on Matrigel (Fig. 7B). The phenotype is similar to that seen when the cells are cultured on SISgel (Fig. 1), except that multiple layers are formed.

## Discussion

When a cancer cell acquires the requisite enabling carcinogenic mutations or a metastatic cell escapes from circulation it is nonetheless in a normal environment that is not permissive of tumor growth due to multiple, redundant controls that function to differentiate and inhibit replication of epithelial cells [1], [2], [7]. Escape from these normal controls likely represents the first, committed step in formation of primary, locally recurrent, and metastatic tumors. We propose that the culture model presented earlier [14] and the xenograft model presented here represent a novel model for investigating the important phenomena of suppression of malignancy and dormancy, and for identifying new therapies to attack metastasis and recurrence at its most vulnerable point.

This model captures several essential elements of suppression and reactivation that are observed clinically. The malignant phenotype of bladder cancer cell lines is suppressed by SISgel *in vitro*, and *in vivo* when implanted with SISgel in flank xenografts. Not only do cells co-injected with SISgel fail to grow immediately as tumors (Fig. 2), but histopathologically they are substantially normalized over the actively growing phenotype (Fig. 3B vs Figs. 3A, C and D). Moreover, most cells are not in the cell cycle and expression of vimentin is greatly reduced over what is seen in actively growing tumors (Fig. 4). This is not just a generalized growth-suppressive effect of SISgel because earlier studies showed that cancer cells seeded onto SISgel have approximately the same rate of proliferation as on Matrigel, and only as they begin to reach confluence does the rate of proliferation slow drastically [13]. Cells co-injected with SISgel can remain suppressed for weeks or even months, long after the SISgel is absorbed, only to emerge as growing tumors, as occurred with about 40% of the implants with SISgel. In the remaining 60%, the cells either remained viable as a green fluorescent spot or disappeared entirely. Three observations suggest that the re-emergence of a tumor is due to a small population of cells rather than to the bulk population. First, re-emergence was positively correlated with the number of cells implanted (Fig. 5B). Second, in two cases the GFP-labeled human cancer cells apparently disappeared, but at a later date following the initial injection the spot reappeared and grew into a tumor. Finally, two re-emergent tumors failed to express GFP, and one was a mosaic of GFP-expressing and non-expressing cells. Because at least 90–95% of the injected cells expressed GPF, the findins suggest the re-emergent tumors derived clonally or from a very small number of cells that either experienced deletion of the GFP gene from its insertion position in the genome or never acquired one initially. In contrast, the immediate, rapid tumor growth in Matrigel suggests survival and growth of the bulk cells under these permissive conditions and is inherent in a significant fraction of the injected cells.

Although other mechanisms can be involved in producing dormancy or suppression [4], [5], [18]–[21], the committed step appears to be escape from the suppressive normal ECM [7], [22]. The findings confirm our previous hypothesis that the ECM exerts a suppressive effect *in vivo* based on loss of malignant properties on SISgel *in vitro* [23]. The suppressive effect of SISgel is not due to the dominant protein, collagen I, or to absence of some factor in SISgel necessary for malignant grown because tumors formed with co-injected collagen I grew nearly as well as they grew when co-injected with Matrigel. Suppression by SISgel appears to be active, not passive, in that some factor in normal extracellular matrix actively suppresses the malignant phenotype, as was suggested more than 15 years ago by Renato Iozzo [1]. Earlier research in our labs suggested that, unlike mammary epithelial cells, α6β4 integrins were not involved [24]. Microarray and proteomic studies implicated complex networks involving TGFβ, cMYC and a series of transcription factors [23], [25], [26]. Because a consistent gene signature could not be identified, it is likely that a protein switch regulates the conversion between the malignant and suppressed phenotypes. The RPPA results suggests that ILK signaling is involved, which is supported by the finding that inhibiting ILK itself prevents invasion. Further research will be required to identify the receptor and detailed signaling mechanisms.

Although the phenomenon of suppression of malignancy is most obviously related to metastasis, phenotypically suppressed cancer cells likely also play an important role in local recurrence. Although some 80–85% of bladder cancers are initially not muscle invasive, the local recurrence rate, up to 70% in some studies [27], with some 15–25% progressing to deadly muscle invasive disease [27] creates a major clinical problem. Recurrences in scars where the margins were tumor-free is most likely not due to failure to detect frank tumor cells in the original histopathologic assessment but is the result of promotion of suppressed cells by the scar microenvironment [28]. Further, finding cells with aberrant DNA content and biomarker expression in morphologically normal urothelium [8] in patients with bladder cancer strongly supports the hypothesis that ECM-modulated suppression of the malignant phenotype occurs *in vivo*. Identifying and targeting such cells could provide a major advance in therapy of bladder cancer. Similar findings in head and neck squamous cell carcinomas suggest the problem may be more general [9].

While the suppression of malignancy by normal ECM likely operates generally and is of importance to the pathobiology of human bladder cancer, understanding the suppression mechanism may identify new targets for control of not only bladder cancer but other tumor types with delayed micrometastases or local recurrence. Both the *in vitro* and *in vivo* models presented here should prove useful in identifying these mechanisms and in providing tractable models for identification of novel drugs to target cancer cells suppressed by the normal extracellular matrix. Preventing delayed metastasis could save thousands of lives per year.

## Materials and Methods

### Fluorescent Protein Expressing Bladder Cancer Cells

The JB-V cell line was derived from the J253 TCC line (ATCC, Manassas, VA) by C. Dinney [16] to be highly metastatic. The J82 line is an aggressive TCC line obtained from the ATCC. Cells were transfected with pLEGFP-C1 retrovirus (Clontech, Mountain View, CA) prepared by co-transfection of the packaging cell line GP2-293 (Clontech, Mountain View, CA) with the pVSV-G vector (Clontech, Mountain View, CA) containing a viral envelope gene. Supernatant from the packaging cells containing the infective virus was collected every 24 hours for 4 days. Fluorescent target cells were made by infecting JB-V and J82 urothelial transitional cell carcinoma cell lines with 1 ml of fresh, virus-containing supernatant/well containing 100,000 target cells along with 8 µg/ml of polybrene (Sigma Aldrich, St. Louis, MO). Each application of viral supernatant was filtered through a 0.4 µm syringe filter before application to target cells. Supernatant was removed and fresh virus-containing media replenished on target cells every 24 hours until 4 changes of media were completed. Virus-containing medium was replaced with Minimum Essential Medium, MEM, (Life Technologies, Carlsbad, CA) containing 1% nonessential amino acids, 1% L-glutamine, 1% sodium pyruvate and 10% fetal calf serum, and cells were allowed to grow to 90% confluence. Stable transfects were selected through sequential sorting and enrichment of fluorescent cells using flow cytometry.

### Growth of Cells on ECM

Gel matrices were made by layering either 0.8 ml of ice cold Matrigel (Becton-Dickinson, Bedford, MA), SISgel, or Type I collagen (BD Biosciences, San Jose, CA) onto polyethylene terephthalate membranes of 6-well cell culture inserts (Falcon, Becton-Dickinson Labware, Franklin Lakes, NJ), which were then allowed to gel at 37°C. SISgel was prepared either by solubilized small intestine submucosa provided to us by Cook Biotech (West Lafayette, IN) or by material we prepared ourselves from decellularized small intestine submucosa using established techniques [29]. Briefly, ground SIS was partially digested with pepsin at pH 2.8 at 4°C for 12 days. This represents an optimization; too little digestion and the material forms clots instead of gelling, and excessive digestion produces material that will not gel. The pepsin was inactivated by raising the pH to 10 and incubating the gel at 4°C for 2 hours, followed by lowering the pH to 4 with 6 N HCl. The product was dialyzed against 10 mM HCl and sterilized with CHCl_3_, which was then dialyzed out against 10 mM HCl. To form a gel, the material was mixed with 1/10^th^ volume of 10X phosphate-buffered saline and a small amount of Phenol Red to assist with pH adjustment. The pH was adjusted to 7.4 with sterile 1 M NaOH using a visual color standard that was checked against a pH meter. Collagen gels were prepared by mixing rat tail collagen, Type I with 1/10^th^ volume of 10X PBS, adjusting the pH to 7.4±0.1, and then layering 0.8 ml onto transwell inserts as described above. Cells were layered onto either Matrigel, SISgel or collagen at a concentration of 5×10^5^ cells/200 µl media, and 2 ml of Dulbecco’s Modified Eagle Medium (DMEM, Life Technologies, Carlsbad, CA) containing 1% penicillin, streptomycin and 10% fetal calf serum were layered beneath the transwell supports in 6-well plates as described [14]. Cultures were grown for 6 days with media changes every two days. Cultures were removed from the transwells, fixed in 1% formalin after overlaying with agarose or SISgel to prevent loss of cells on cutting of sections. Sections (5 µm) were stained with hematoxylin and eosin. The role of Integrin Linked Kinase (ILK) signaling was confirmed by adding the ILK inhibitor, QLT0267, (kindly provided by S. Dedhar) to the culture medium of cells grown on Matrigel and SISgel at a concentration of 10 µM and determining the phenotype from stained sections.

Cultured cells grown for 3 days were isolated from the matrices as follows. Cells grown on Matrigel were gently washed with Hank’s Balanced Salts Solution, (HBSS, Life Technologies, Carlsbad, CA), then coarsely minced gels were incubated with 1 ml of dispsase (BD Biosciences, San Jose, CA) at 37°C until the gel dissolved. Cells were harvested from SISgel or collagen by washing with HBSS then incubating coarsely minced gels with 1 ml of Collagenase IV, 450 U/ml, (Life Technologies, Carlsbad, CA) at 37°C until the gel dissolved. Liberated cells were centrifuged to a pellet, washed twice with HBSS, then resuspended with DMEM. Cells grown on plastic were trypsinized and resuspended in DMEM.

### Growth of Cells Flank Xenograft Model

This study was carried out in strict accordance with the recommendations in the Guide for the Care and Use of Laboratory Animals of the National Institutes of Health. The protocol was approved by the Committee on the Ethics of Animal Experiments of the Oklahoma University Health Sciences (Protocols 06-140 and 09-041). All efforts were made to minimize suffering. Cells resuspended in 100 µl DMEM/flank were mixed with an equal volume of cold SISgel, Matrigel or collagen, type I. The mixture of gel and cells prepared as described in the previous section was immediately injected subcutaneously into either the right or left flank of a 5-week old nude mouse, nu/nu-nuBR, (Charles River Laboratories, Wilmington, MA). Caliper measurements of tumor size were taken every week for the length of the study as were fluorescent images. Tumors were allowed to grow until the animals developed signs of distress. In case no tumors developed, experiments were terminated at 120 days. Labeled cells were visualized with the Lightool’s LT-9900 system with the EGFP filter set of 470 nm excitation filter and 515 nm viewing filter and captured with a cooled CCD camera (Lightools, Encinitas, CA). The area and intensity of the tumor was measured using Adobe Photoshop by first selecting the tumor area, then counting pixels above a threshold selected to eliminate background, non-tumor areas. The integrated intensity was calculated by multiplying the average intensity of detected pixels by the number of pixels detected. Images were scored as “growing” if the integrated intensity increased by more than 10% over two consecutive measurements. H&E stained sections were examined by a board-certified pathologist (SDK).

### Histology and Immunohistochemistry

Tumors and suppressed “spots” were excised from animals after euthanasia. The excised tissue was preserved in formalin, mounted in paraffin, sectioned into 5 µm sections and placed on slides. One set was stained with hematoxylin and eosin by standard techniques and examined by a board-certified animal pathologist (SDK). Another set was labeled for immunohistochemical analysis following antigen retrieval using a Retreiver 2100 (PickCell Lab) and R-Buffer A (Electron Microscopy Sciences, #62706-10). After a 20 min heating cycle, the machine was allowed to cool at least two hours before removing the slides. The slides were rinsed in de-ionized water for 5 min. The tissues were circle with pap pen, then blocked for 20 min with normal goat serum (kit #AK-5001, Vector Labs Inc). The slides were incubated with primary antibody for 45 min at room temp (1∶100 dilutions, vimentin (cell signaling #5741), ki-67 (Abcam #16667), and washed 3×3 min. with TBS pH 7.5. The slides were incubated with biotinylated anti-Rabbit IgG (kit #AK-5001), then rinsed with TBS 3×3 min. The slides were incubated for 30 min with ABC-AP reagent (kit #AK-5001), washed 3×3 min with TBS. The slides were incubated for 14 min with Vector Blue AP Substrate (SK-5300, Vector Labs), rinsed for 5 min with TBS, and 5 min with de-ionized water. The nuclei were counterstained with 0.1% Nuclear Fast Red. The slides were rinsed 5 min with de-ionized water, 5 dips in each of three changes of 95% ethanol, then 5 dips in each of three changes of 100% ethanol. The slides were air dried for 15 min, dipped in Histoclear, a drop of MM24 mounting medium was added (Surgipath Medical Industries, # 01120) and a cover slip was placed over the tissue.

### Microarray Analysis of Gene Expression by Matrix

Total RNA was isolated from J82 and JBV cells grown on Matrigel and SISgel and liberated as described above. Triplicate samples, each from a different six-well transwell, were prepared to use as biological replicates. The RNA was isolated using a filter method with the “QuickGene-Mini80” device and reagents (FujiFilm Life Sciences marketed by AutoGen, Holliston, MA). http://www.autogen.com/assets/pdf/White%20Paper%20-%20QuickGene.pdf. RNA integrity was assessed using the Agilent (Agilent Technologies, Santa Clara, CA) microelectrophoresis system. The RIN was >9.0 for all the RNA preparations. The RNAs were converted to sense cDNA and hybridized to Affymetrix GeneChip Human Gene 1.0 ST Arrays (Affymetrix, Inc., according to the manufacturer's instructions. This array has 764,885 total 23-mer probes, which correspond to 28,869 annotated gene level probe sets and covers 99% of the sequences present in the RefSeq database.

### Reversed Phase Protein Array (RPPA) Analysis of Protein Expression

Cell pellets were lysed by adding 250 ul of lysis buffer and incubating on ice for 20 min with mixing every 5 min. Lysis buffer was obtained from MD Anderson RPPA Core containing: 1% Triton X-100, 50 mM HEPES, pH 7.4, 150 mM NaCl, 1.5 mM MgCl_2_, 1 mM EGTA, 100 mM NaF, 10 mM Na pyrophosphate, 1 mM Na_3_VO_4_, 10% glycerol, containing freshly added protease inhibitor cocktail (Sigma #P2714) and phosphatase inhibitors (Sigma). Lysates were centrifuged at 13,000 rpm for 5 minutes at 4°C and the supernatant was collected. Protein concentration was determined with Bradford assay (Bio-Rad) and adjusted to 1.5 ug/ml. Then 60 µg lysate was mixed with 4° SDS sample buffer without bromophenol blue (4× buffer was from MD Anderson: 40% Glycerol, 8% SDS, 0.25 M Tris-HCL, pH 6.8, 10% 2-mercaptoehtanol) and boiled for 5 minutes, the samples were stored at –80°C and shipped with dry ice to the MD Anderson RPPA Core. Lysates were two-fold-serial diluted for 5 dilutions (from undiluted to 1∶16 dilution) and arrayed on nitrocellulose-coated slide in 11×11 format. Samples were probed with antibodies by CSA amplification approach and visualized by DAB colorimetric reaction. Table S1 lists the characteristics of all the antibodies used in this study. Slides were scanned on a flatbed scanner to produce 16-bit tiff image, and spots from tiff images were identified and the density was quantified by MicroVigene. Relative protein levels for each sample were determined by interpolation of each dilution curves from the “standard curve” of the slide (antibody). After deletion of spots and antibodies that failed the QC test, a total of 207 protein values were reported, of which 17 were duplicates.

### Analysis of High-Dimension Microarray and RPPA Data

Raw microarray data were processed using Affymetrix Expression Console version 1.1 (http://www.affymetrix.com/browse/level_seven_software_products_only.jsp?productId=131414&categoryId=35623#) to extract gene level intensities using the RMA method. The subsequent analysis was performed using the R statistical environment (http://www.R-project.org) and BioConductor packages [30]. Quality control of the data included a comparison of boxplots of gene expression level among the conditions, and hierarchical clustering of condition-specific gene expression profiles to identify potential outliers. The data were quantile normalized and deposited in the GEO database along with raw.CEL files (Accession number GSE35947). All data conform with the Minimal Information About a Microarray Experiment (MIAME) guidelines. Matrix-specific combined gene/protein expression profiles were compared using the *limma* [31] package. The Benjamini-Hochberg false discovery rate (FDR) correction for multiple testing [32] was applied, along with 2-fold change cutoff. Gene set enrichment analysis (GSEA) method [33] was used to analyze differentially expressed genes ranked by their fold change. Briefly, GSEA walks down the list of pre-ranked genes and calculates enrichment of gene groups in a given functional category. Functional categories containing genes consistently up- or downregulated are further assessed by a permutation method. GSEA assesses enriched categories in either phenotype by starting from either up- or downregulated genes. In this study, we considered enrichment of the genes in KEGG canonical pathways [34] and all gene ontology categories [35]. Further investigation of functional significance of differentially expressed genes/proteins was carried out by Ingenuity Pathway Analysis (IPA; Ingenuity Systems, Redwood City, CA).

## Supporting Information

Table S1 **Characteristics of antibodies used to probe cell lysates by RPPA analysis.**
(DOCX)
