# Supplementary material for: Suppression and Activation of the Malignant Phenotype by Extracellular Matrix in Xenograft Models of Bladder Cancer: A Model for Tumor Cell “Dormancy”
Source: PLoS One. 2013 May 24;8(5):e64181. doi: 10.1371/journal.pone.0064181 (PMC3663841; doi:10.1371/journal.pone.0064181)
Supplement: Table S1 — Characteristics of antibodies used to probe cell lysates by RPPA analysis. (DOCX) [file pone.0064181.s001.docx]

Table S1: Characteristics of antibodies used to probe cell lysates by RPPA analysis

| **Ab Name** | **Company** | **Catalog #** | **Ab ID** | **Species** | **Validation Status*** |
| --- | --- | --- | --- | --- | --- |
| 14-3-3 Beta | Santa Cruz | sc-628 | 882.1 | Rabbit | Use with Caution |
| 14-3-3 Zeta | Santa Cruz | sc-1019 | 883.1 | Rabbit | Use with Caution |
| 4EBP1 | CST | 9452 | 2.8 | Rabbit | Validated |
| 4EBP1 pS65 | CST | 9456 | 3.1 | Rabbit | Validated |
| 4EBP1 P-T37/T46 | CST | 9459 | 6.4 | Rabbit | Validated |
| 4EBP1 pT70 | CST | 9455 | 8.6 | Rabbit | Use with Caution |
| 53BP1 | CST | 4937 | 985.1 | Rabbit | Use with Caution |
| ACC S79 | CST | 3661 | 13.4 | Rabbit | Validated |
| ACC1 | Epitomics | 1768-1 | 14.1 | Rabbit | Use with caution |
| AIB-1 | BD Biosciences | 611105 | 711.1 | Mouse | Validated |
| AKT | CST | 9272 | 21.13 | Rabbit | Validated |
| AKT S473 | CST | 9271 | 23.10 | Rabbit | Validated |
| AKT T308 | CST | 9275 | 25.11 | Rabbit | Validated |
| Akt1 | CST | 2967 | 20.3 | Mouse | Use with Caution |
| Akt2 | CST | 2964 | 20.3 | Rabbit | Use with Caution |
| AMPK | CST | 2532 | 39.4 | Rabbit | Use with caution |
| AMPK T172 | CST | 2535 | 40.6 | Rabbit | Validated |
| Annexin | Invitrogen 71-3400 | 71-3400 | 795.1 | Rabbit | Validated |
| AR | Epitomics | 1852-1 | 756.1 | Rabbit | Validated |
| ATM | Abcam | ab32420 | 54.1 | Rabbit | Use with caution |
| ATM pS1981 | Rockland | 200-301-400 | 989.1 | Mouse | Use with Caution |
| ATR | CST | 2790 | 900 | Rabbit | Use with caution |
| ATR pS428 | CST | 2853 | 901.3 | Rabbit | Use with Caution |
| BAD pS112 | CST | 9291 | 63.1 | Rabbit | Use with caution |
| BAD pS155 | CST | 9297 | 68.20 | Rabbit | Use with Caution |
| Bak | Epitomics | 1542-1 | 71.1 | Rabbit | Use with caution |
| BAX | CST | 2772 | 73.3 | Rabbit | Use with caution |
| B-Catenin (P-S33/S37/T41) | CST | 9561 | 77.3 | Rabbit | Use with caution |
| Bcl-2 | Dako M0887 | Dako M0887 | 80.1 | Mouse | Validated |
| Bcl-X | Epitomics | 1018-1 | 84.1 | Rabbit | Use with caution |
| Bcl-xL | CST | 2762 | 85.5 | Rabbit | Use with caution |
| Beclin | Santa Cruz | sc-10086 | 87.1 | Goat | Validated |
| Bid | Epitomics | 1008-1 | 88.1 | Rabbit | Use with caution |
| BIM | Epitomics | 1036-1 | 90.1 | Rabbit | Validated |
| BRCA2 | CST | 9012 | 761.1 | Rabbit | Use with Caution |
| Cadherin-E | CST | 4065 | 209.2 | Rabbit | Validated |
| Cadherin-N | CST | 4061 | 452.1 | Rabbit | Validated |
| Cadherin-P | CST | 2130 | 509.1 | Rabbit | Use with caution |
| Casein kinase I ε | BD | 610445 | 933.1 | Mouse | Use with Caution |
| Caspase 3 Active | Epitomics | 1476-1 | 108.1 | Rabbit | Use with caution |
| Caspase 7 cleaved Asp198 | CST | 9491 | 109.6 | Rabbit | Use with caution |
| Caspase 9 (Cleaved) | CST | 9491 | 109 | Rabbit | Use with Caution |
| Catechol-O-methyltransferase (COMT) | SDI--GA1671 | SDI--GA1671 | 112.1 | Rabbit | Validated |
| Catenin Beta | CST | 9562 | 75.3 | Rabbit | Validated |
| Caveolin 2 | BD | 610684 | 116.1 | Mouse | Use with Caution |
| Caveolin1 | CST | 3238 | 114.1 | Rabbit | Validated |
| CD20 | Epitomics | 1632-1 | 125.1 | Rabbit | Use with Caution |
| CD31 | Dako | M0823 | 127.1 | Mouse | Validated |
| CD86 | Abcam | ab53004 | 1013.2 | Rabbit | Use with Caution |
| CDC2 | CST | 9112 | 1007.5 | Rabbit | Validated |
| CDK4 | BD Biosciences | 610147 | 772.1 | Mouse | Use with Caution |
| Chk1 | CST | 2345 | 145.10 | Rabbit | Use with caution |
| Chk1 pSer345 | CST | 2348 | 903.70 | Rabbit | Use with caution |
| Chk2 (1C12) | CST | 3440 | **146.1** | Mouse | Use with caution |
| Chk2 pThr68 | CST | 2197 | **147.2** | Rabbit | Use with caution |
| cJun P-S73 | CST | 9164 | 155.5 | Rabbit | Use with caution |
| Claudin7 CLDN7 | Novus | NB100-91714 | 852.1 | Rabbit | Validated |
| Cofilin | CST | 3312 | 954.7 | Rabbit | Use with Caution |
| Collagen VI | Santa Cruz | SC-20649 | 171.1 | Rabbit | Validated |
| Connexin | SDI--GA1716 | SDI--GA1716 | 174.1 |  | Validated |
| Cox-2 | Epitomics | 2169-1 | 755.1 | Rabbit | Use with caution |
| CREB | CST | 9197 | 181.7 | Rabbit | Use with Caution |
| CUL-2 | BD | 610778 | 939.1 | Mouse | Use with Caution |
| Cyclin B1 | Epitomics | 1495-1 | 192.1 | Rabbit | Validated |
| Cyclin D1 | Santa Cruz | SC-718 | 194.1 | Rabbit | Validated |
| Cyclin E1 | Santa Cruz | SC-247 | 201.1 | Mouse | Validated |
| Cyclin E2 | Epitomics | 1142-1 | 202.1 | Rabbit | Use with caution |
| Cyclin E4 | Santa Cruz | SC-25303 | 199.1 | Mouse | Use with Caution |
| Cytokeratin 17 | Lab Vision | MS-489-S0 | 386.1 | Mouse | Use with Caution |
| Cytokeratin 18 | Lab Vision | MS-142-P0 | 868.1 | Mouse | Use with Caution |
| Cytokeratin 19 | Lab Vision | MS-198-P0 | 869.1 | Mouse | Use with Caution |
| DJ-1 | Abcam | ab76008 | 891.10 | Rabbit | Use with caution |
| Dvl3 | CST | 3218 | 940.1 | Rabbit | Use with Caution |
| EEA1 | BD | 610457 | 973.1 | Mouse | Use with Caution |
| eEF2 | CST | 2332 |  | Rabbit | Validated |
| eEF2K | CST | 3692 |  | Rabbit | Validated |
| EGFR | Santa Cruz | SC-03 | 215.2 | Rabbit | Use with caution |
| EGFR Y1173 | Epitomics | 1124 | 221 | Rabbit | Use with caution |
| EGFR Y992 | CST | 2235 | 222.4 | Rabbit | Validated |
| eIF4E | CST | 9742 | 722.3 | Rabbit | Validated |
| EIG121 | SDI | SDI | 874.1 |  | Use with caution |
| Elk-1 S383 | CST | 9181 | 228.3 | Rabbit | Use with Caution |
| En1 | SDI 2104 | SDI 2104 |  |  | Validated |
| ER-a S118 | Epitomics | 1091-1 | 241.1 | Rabbit | Validated |
| ERCC1 | Lab Vision | MS-671-PO | 247.1 / | Mpose | Use with caution |
| ETV6 | SDI--GA2101 | SDI--GA2101 | 872.1 |  | Use with Caution |
| Ezrin pThr567 /Radixin (Thr564)/Moesin (Thr558) | CST | 3141 | 895.11 | Rabbit | Use with Caution |
| Ezrin pTyr353 | CST | 3144 | 942.2 | Rabbit | Use with Caution |
| FAK | Epitomics | 1700-1 | 252.2 | Rabbit | Use with caution |
| FANCD2 | Abcam | ab12450 | 986.10 | Mouse | Use with Caution |
| FGF Receptor 3 (D2G7E) | CST | 3163 | 958.1 | Rabbit | Use with Caution |
| Fibronectin | Epitomics | 1574-1 | 262.10 | Rabbit | Use with caution |
| FOX03a | CST | 9467 | 269.4 | Rabbit | Use with caution |
| FOX03a S318/321 | CST | 9465 | 270.1 | Rabbit | Use with caution |
| Gata3 | BD Biosciences | 558686 | 764.1 | Mouse | Validated |
| Gelsolin | SDI--GA1718 | SDI--GA1718 | 276.1 |  | Validated |
| GSK3 S21/S9 | CST | 9331 | 285.12 | Rabbit | Validated |
| GSK3-Beta | Santa Cruz | SC-7291 | 284.2 | Mouse | Validated |
| Hamartin/TSC1 | CST | 4906 | Funda-Meric | Rabbit | Validated |
| hCRT1 | Kuo's Lab UCSF | Kuo's Lab UCSF | Mills |  | Validated |
| HER2 pY1248 | Upstate (Millipore) | 06-229 | 299.1 | Rabbit | Validated |
| HER2/ErbB2 pTyr877 | CST | 2241 | 300.2 | Rabbit | Use with Caution |
| Her3 | Lab Vision | MS-201 | 301.1 | Mouse | Use with Caution |
| Heregulin | CST | 2573 | 890.1 | Rabbit | Validated |
| Histone H2A.X pSer139 | Upstate (Millipore) | 05-636 | 960.1 | Mouse | Use with Caution |
| HMGA1 | SDI #2221 | SDI #2221 | Mills |  | Validated |
| HSP27 | CST | 2402 | 321.2 | Mouse | Use with caution |
| Hsp27 pS82 | CST | 2401 | 323.8 | Rabbit | Use with Caution |
| HSP70 | CST | 4872 | 325.2 | Rabbit | Use with caution |
| HSP90 | CST | 4874 | 326.7 | Rabbit | Use with Caution |
| IGFBP2 | CST | 3922 | 335.1 | Rabbit | Validated |
| IGFR1b | CST | 3027 | 336.1 | Rabbit | Use with caution |
| INPP4B | Santa Cruz | SC-12318 | 912.1 | Rabbit poly | Use with caution |
| IRS-1 | Upstate (Millipore) | 06-248 | 802.1 | Rabbit | Validated |
| Jnk2 | CST | 4672 | 380.1 | Rabbit | Use with caution |
| Kit-c | Epitomics | 1522 | 157.00 | Rabbit | Validated |
| K-RAS | Santa-Cruz | sc-30 (F234) | none | Mouse | Use with caution |
| Ku80 | CST | 2180 | 904.10 | Rabbit | Use with caution |
| Lamin A/C | CST | 2032 | 395.3 | Rabbit | Use with Caution |
| LKB1 | Abcam | ab15095 | 399.00 | Mouse |  |
| Malt1 | SDI | SDI | 873.1 | Rabbit | Use with Caution |
| MAPK P-T202/204 | CST | 4377 | 405.3 | Rabbit | Validated |
| MAPK-p44/42 pThr202/Tyr204 | CST | 9106 | 403.30 | Rabbit | Use with Caution |
| Mcl1 | BD Biosciences | 559027 | 410.1 | Mouse | Use with Caution |
| MEK1 | Epitomics | 1235-1 | 417.1 | Rabbit | Validated |
| MEK1 pS217/221 | CST | 9121 | 419.15 | Rabbit | Validated |
| Met | CST | 3127 | 726.3 | Mouse | Use with Caution |
| Met pTyr1234/1235 | CST | 3129 | 727.4 | Rabbit | Use with Caution |
| MIG-6 | Sigma | WH0054206M1 |  | Mouse | Validated |
| MME | Santa Cruz | sc-52994 | Mills | Mouse | Use with caution |
| Mre11(31H4) | CST | 4847 | 440.1 | Rabbit | Use with caution |
| MSH2 | CST | 2850 | 905.1 | Mouse | Use with caution |
| MSH6 | SDI | SDI |  | Rabbit | Use with caution |
| mTOR | CST | 2983 | 444.1 | **Rabbit** | Validated |
| mTOR S2448 | CST | 2971 | 446.11 | **Rabbit** | Validated |
| Myc | CST | 9402 | 161.2 / Kornblau | Rabbit | Use with caution |
| NCKIPSD | SDI #2117 | SDI #2117 | Mills | Rabbit | Validated |
| NF2 | SDI #2271 | SDI #2271 | Mills | Rabbit | Use with caution |
| NF-kB p65 | CST | 3033 | 457.00 | Rabbit | Use with caution |
| Notch 1 | CST | 3268 | Mills | Rabbit | Validated |
| Notch3 | Santa Cruz | sc-5593 | 767.1 | Rabbit | Use with caution |
| p15 INK4B | CST | 4822 | 464.1 | Rabbit | Use with Caution |
| p21 | Santa Cruz | SC-397 | 470 | Rabbit | Use with caution |
| p27 | Epitomics | 1591-1 | 897.1 | Rabbit | Validated |
| p27 pT157 | R&D | AF1555 | 842.1 | Rabbit | Use with caution |
| p27 pT198 | Abcam | ab64949 | 878.1 | Rabbit | Validated |
| p38 / MAPK | CST | 9212 | 478.10 | Rabbit | Use with caution |
| p38 T180/182 | CST | 9211 | 479.15 | Rabbit | Validated |
| p53 | CST | 9282 | 481.3 | Rabbit | Validated |
| p70S6K | Epitomics | 1494-1 | 493.1 | Rabbit | Validated |
| p70S6K T389 | CST | 9205 | 494.7 | Rabbit | Validated |
| p90 RSK P-T359/S363 | CST | 9344 | 770.2 | Rabbit | Use with caution |
| Parp 1 cleaved p85 | Epitomics | 1074-1 | 502.1 | Rabbit | Use with Caution |
| PARP cleaved | CST | 9546 | 501.10 | Mouse | Use with caution |
| Paxillin | Epitomics | 1500-1 | 505.1 | Rabbit | Validated |
| PCNA | Abcam | ab29 | 511.1 | Mouse | Validated |
| PDCD4 | Rockland | 600-401-965 | 816.10 | Rabbit | Use with Caution |
| PDK1 | CST | 3062 | 515.5 | Rabbit | Validated |
| PDK1 P-S241 | CST | 3061 | 516.7 | Rabbit | Validated |
| Pea15 | CST | 2780 | 1017.1 | Rabbit | Validated |
| Pea15 pS116 | Invitrogen | 44-836G | 1018.1 | Rabbit | Validated |
| Phospho-Cofilin (Ser3) (77G2) | CST | 3313 | 955.1 | Rabbit | Use with Caution |
| PI3K P110a | CST | 4255 | 808.10 | Rabbit | Use with caution |
| PI3K-p85 | Upstate (Millipore) | 06-195 | 523.3 or 523.4 | Rabbit | Validated |
| PKC (pan) | CST | 9371 | Funda-Meric | Rabbit | Validated |
| PKC S657 | Upstate (Millipore) | 06-822 | 530.2 | Rabbit | Validated |
| PKCa | Upstate (Millipore) | 05-154 | 529.1 | Mouse | Validated |
| PKM2 | CST | 4053S | 1025.1 | Rabbit | Use with Caution |
| PLCr2 | CST | 3874 | 1030.4 | Rabbit | Use with Caution |
| PLCγ1 | CST | 2822 | 543.1 | Rabbit | Use with Caution |
| PLK | CST | 4513 | 754.40 | Rabbit | Use with caution |
| PR | Epitomics | 1483-1 | 549.1 | Rabbit | Validated |
| Pras40 pT246 | Biosource | 441100G | 739.1 | Rabbit | Validated |
| PTCH | SDI #2113 | 2113.00.02 |  | Rabbit | Use with caution |
| PTEN | CST | 9552 | 566.3 | Rabbit | Validated |
| PTEN (138G6) | CST | 9559 | 567.7 | Rabbit | Use with Caution |
| PTEN pSer380/Thr382/383 | CST | 9554 | 573.5 | Rabbit | Use with Caution |
| Rab11 | BD Biosciences | 610656 | 575.1 | Mouse | Use with Caution |
| Rab25 | Covance Custom | Covance Custom | **577.1** | Rabbit | Use with caution |
| Rab5 | BD Biosciences | 610724 | 578.1 | Mouse | Use with Caution |
| Rad50 | Millipore | 05-525 | 987.1 | mouse | Use with caution |
| Rad51 | Chem Biotech | na 71 | 579.30 | Mouse | Use with caution |
| Raf-A pS299 | CST | 4431 | Mills | Rabbit | Use with caution |
| Raf-B | Santa Cruz | sc-5284 | 96.1 | Mouse | Use with caution |
| Raf-C | Millipore | 05-739 | 803.00 | Rabbit | Validated |
| Raf-c pS388 | CST | 9427 | 179.40 | Rabbit | Use with caution |
| Rb (4H1) | CST | 9309 | 552.7 | Mouse | Validated |
| Rb pS807/811 | CST | 9308 | 557.9 | Rabbit | Validated |
| Rheb | R&D | MaB3426 | 847.1 | Mouse | Use with Caution |
| S6 S235/236 | CST | 2211 | 600.8 | Rabbit | Validated |
| S6 S240/244 | CST | 2215 | 601.4 | Rabbit | Validated |
| SAPK/JNK pThr183/Tyr185 | CST | 9251 | 378.14 | Rabbit | Use with Caution |
| SCD | Santa Cruz | sc-58420 | Funda-Meric |  | Validated |
| SEK1/MKK4 | CST | 9152 | 429.2 | Rabbit | Use with Caution |
| SF2/ASF | Zymed/Invitrogen | 32-4500 | Funda-Meric |  | Validated |
| Shc pY317 | CST | 2431 | Mills | Rabbit | Use with caution |
| Smad3 | Epitomics | 1735-1 | 796.1 | Rabbit | Validated |
| Smad-5 pSer463/465 | Epitomics | 2224-1 | 799.1 | Rabbit | Use with Caution |
| Snail | CST | 3895 | 616.10 | Mouse | Use with caution |
| Src | Upstate (Millipore) | 05-184 | 621.2 | Mouse | Validated |
| Src (36D10) | CST | 2109 | 620.4 | Rabbit | Use with Caution |
| Src P-Y527 | CST | 2105 | 626.5 | Rabbit | Validated |
| Src Y416 | CST | 2101 | 623.11 | Rabbit | Use with caution |
| Stat3 P-S705 | CST | 9131 | 637.6 | Rabbit | Validated |
| Stat5 | Epitomics | 1289-1 | 638.1 | Rabbit | Validated |
| Stathmin | Epitomics | 1972-1 | 718.1 | Rabbit | Validated |
| Survivin | CST | 2802 | 643.2 | Mouse | Use with Caution |
| Syk | Santa Cruz | sc-1240 | 1033.1 | Mouse | Use with Caution |
| Tau | Upstate (Millipore) | 05-348 | 646.1 | Mouse | Use with caution |
| Taz | Abcam | ab3961 | 778.1 | Rabbit | Validated |
| Taz P-Ser79 | Santa Cruz | sc-17610 | 779.0 | Rabbit | Use with caution |
| Telomerase | SDI--GA1706 | SDI--GA1706 | 647.1 | Rabbit | Use with caution |
| TopoII | Abcam | ab45175 | 794.1 | Discontinued | Use with Caution |
| Topoisomerase II a pThr1343 | Epitomics | 1871-1 | 666.1 | Rabbit | Use with Caution |
| Transglutaminase | Lab Vision | MS-224 | 908.1 | Mouse | Validated |
| TRFC | SDI #2250 | SDI #2250 | Mills |  | Validated |
| Tuberin/TSC2 | Epitomics | 1613-1 | 670.1 | Rabbit | Use with caution |
| Tubulin alpha | CST | 2125 | 950.2 | Rabbit | Use with Caution |
| Vasp | CST | 3112 | 678.2 | Rabbit | Use with caution |
| VEGFR2 | CST | 2479 | 688.4 | Rabbit | Validated |
| XBP1 | Santa Cruz | sc32136 | Mills | Goat | Use with Caution |
| Xiap | CST | 2042 | 699.6 | Rabbit | Use with caution |
| XRCC1 | CST | 2735 | 906.10 | Rabbit | Use with caution |
| Y Box Binding Protein 1 | SDI--GA1725 | 1725.00.02 | 700.1 | Rabbit | Validated |
| YAP | sc-15407 | sc-15407 | 780.0 | Rabbit | Validated |
| YAP pS127 | CST | 4911 | 782.10 | Rabbit | Use with caution |
| YB1 pS102 | CST | 2900 | 835.1 | Rabbit | Validated |
